# Perceptual Doping: An Audiovisual Facilitation Effect on Auditory Speech Processing, From Phonetic Feature Extraction to Sentence Identification in Noise

**DOI:** 10.1097/AUD.0000000000000616

**Published:** 2019-02-27

**Authors:** Shahram Moradi, Björn Lidestam, Elaine Hoi Ning Ng, Henrik Danielsson, Jerker Rönnberg

**Affiliations:** 1 Linnaeus Centre HEAD, Swedish Institute for Disability Research, Department of Behavioral Sciences and Learning, Linköping University, Linköping, Sweden; 2 Department of Behavioral Sciences and Learning, Linköping University, Linköping, Sweden; 3 Oticon A/S, Smørum, Denmark.

**Keywords:** Audiovisual speech facilitation, Consonants, Perceptual doping, Vowels, Sentence-in-noise

## Abstract

**Objective:**

We have previously shown that the gain provided by prior audiovisual (AV) speech exposure for subsequent auditory (A) sentence identification in noise is relatively larger than that provided by prior A speech exposure. We have called this effect “perceptual doping.” Specifically, prior AV speech processing dopes (recalibrates) the phonological and lexical maps in the mental lexicon, which facilitates subsequent phonological and lexical access in the A modality, separately from other learning and priming effects. In this article, we use data from the n200 study and aim to replicate and extend the perceptual doping effect using two different A and two different AV speech tasks and a larger sample than in our previous studies.

**Design:**

The participants were 200 hearing aid users with bilateral, symmetrical, mild-to-severe sensorineural hearing loss. There were four speech tasks in the n200 study that were presented in both A and AV modalities (gated consonants, gated vowels, vowel duration discrimination, and sentence identification in noise tasks). The modality order of speech presentation was counterbalanced across participants: half of the participants completed the A modality first and the AV modality second (A1–AV2), and the other half completed the AV modality and then the A modality (AV1–A2). Based on the perceptual doping hypothesis, which assumes that the gain of prior AV exposure will be relatively larger relative to that of prior A exposure for subsequent processing of speech stimuli, we predicted that the mean A scores in the AV1–A2 modality order would be better than the mean A scores in the A1–AV2 modality order. We therefore expected a significant difference in terms of the identification of A speech stimuli between the two modality orders (A1 versus A2). As prior A exposure provides a smaller gain than AV exposure, we also predicted that the difference in AV speech scores between the two modality orders (AV1 versus AV2) may not be statistically significantly different.

**Results:**

In the gated consonant and vowel tasks and the vowel duration discrimination task, there were significant differences in A performance of speech stimuli between the two modality orders. The participants’ mean A performance was better in the AV1–A2 than in the A1–AV2 modality order (i.e., after AV processing). In terms of mean AV performance, no significant difference was observed between the two orders. In the sentence identification in noise task, a significant difference in the A identification of speech stimuli between the two orders was observed (A1 versus A2). In addition, a significant difference in the AV identification of speech stimuli between the two orders was also observed (AV1 versus AV2). This finding was most likely because of a procedural learning effect due to the greater complexity of the sentence materials or a combination of procedural learning and perceptual learning due to the presentation of sentential materials in noisy conditions.

**Conclusions:**

The findings of the present study support the perceptual doping hypothesis, as prior AV relative to A speech exposure resulted in a larger gain for the subsequent processing of speech stimuli. For complex speech stimuli that were presented in degraded listening conditions, a procedural learning effect (or a combination of procedural learning and perceptual learning effects) also facilitated the identification of speech stimuli, irrespective of whether the prior modality was A or AV.

## Abstract 2

Supplemental Digital Content is available in the text.

## INTRODUCTION

Speech perception is inherently multisensory rather than unisensory. Seeing facial gestures in face-to-face communication facilitates the identification of speech stimuli compared with auditory (A) communication (Sumby & Pollack 1954; Erber 1969; Sommers et al. 2005; Moradi et al. 2016). In addition, cross-modal studies have shown that prior visual-only (V) exposure to speech stimuli subsequently improved the A identification of speech stimuli (e.g., Rosenblum et al. 2007; Wu et al. 2013; see the review by Rosenblum et al. 2017, for cross-modal interactions in speech perception). Furthermore, recent studies have revealed that audiovisual (AV) relative to A presentation reduces the cognitive demands (effort) required for the identification of speech stimuli in degraded listening conditions (such as in background noise or in people with hearing loss; Mishra et al. 2013; Moradi et al. 2013; Frtusova & Phillips 2016; Moradi et al. 2017a). Moreover, AV speech training has been shown to be better than A speech training in improving the A identification of speech stimuli (Kawase et al. 2009; Bernstein et al. 2013; Alghamdi et al., Reference Note 1). According to Shams and Seitz (2008), the human brain has evolved to operate, process, and learn ideally in multisensory rather than unisensory conditions, as the external environment presents our senses with multisensory cues regarding a given event.

The abovementioned advantages of AV over A speech presentations were apparent when researchers simultaneously compared AV with A speech perception (or training) in terms of accuracy, speed of identification, and cognitive demands. Yet, little is known about how prior AV speech exposure affects the subsequent identification of speech stimuli.

Moradi et al. (2013), using the gating paradigm (Grosjean 1980), tried to estimate the extent to which AV relative to A presentation would facilitate the identification of different types of speech stimuli (consonants, words, and sentences). In the gating paradigm, participants are presented with successive fragments of a given speech token (e.g., a word), and their task is to correctly identify the speech token using as few fragments of the token as possible, as assessed in terms of isolation points (IPs; i.e., the shortest time from the onset of a speech token that is required for correct identification).

An incidental finding by Moradi et al. (2013) was that those participants who were first exposed to AV gated speech stimuli subsequently performed better in a test of purely A sentence identification in noise (Hearing in Noise Test [HINT]; Hällgren et al. 2006) than those who were first exposed to A gated speech stimuli. In addition, the speakers of the AV gated speech stimuli and subsequent A HINT were not the same, indicating that the AV facilitation effect on subsequent A sentence identification in noise was independent of the idiosyncrasy of speakers.

Lidestam et al. (2014) conducted a randomized control study to evaluate whether the findings of Moradi et al. (2013) represented a genuine effect. In their study, participants were divided into three groups: a group who received gated AV speech training with consonants and words, a group who received gated A speech training with consonants and words, and a control group who just observed a movie clip. Their HINT scores were obtained before and after the training. The speakers of the speech stimuli in the training materials (gated A and AV speech tasks and the movie clip) and the A HINT task were not the same. The results showed that only the participants who received gated AV speech training subsequently performed better in the HINT, but not the other two groups.

Moradi et al. (2017b) used the gated AV and A speech materials used in Lidestam et al. (2014), but recruited elderly hearing aid users, to evaluate the efficiency of gated AV speech training and the maintenance of training-related improvements in subsequent A HINT performance. Their results generalized the findings by Lidestam et al. by showing that short AV speech exposure (of around 35 min) to gated consonants and words promptly improved A HINT performance (post-test training versus pretest training); furthermore, this improvement was maintained 1 month after the AV speech training (1-month follow-up versus pretest training).

Moradi et al. (2017b) coined the term “perceptual doping” to explain how prior AV speech exposure subsequently facilitates the A identification of speech stimuli. According to this hypothesis, prior AV speech exposure can recalibrate (or retune) phonological and lexical maps in the mental lexicon, such that the maps become more distinct and easily accessible *without effort*. These recalibrated maps are saved and subsequently used to ease the A mapping of incoming speech signals with their *recalibrated* (by prior AV exposure) corresponding phonological and lexical representations in the mental lexicon.

Here, one may argue that the AV facilitation effect on subsequent improvement in processing speech stimuli in fact represents perceptual learning or perceptual priming or another similar perceptual facilitation effect (e.g., procedural learning or an AV recalibration effect). Perceptual priming refers to the enhanced performance that occurs following prior exposure (in whole or in part) to a given target item (Tulving & Schacter 1990; Schacter & Buckner 1998). Similarly, perceptual learning refers to an improvement in responding to a stimulus achieved through practice or repeated exposure to that stimulus (Gibson 1963; Goldstone 1998). Procedural learning also refers to enhanced performance in a task achieved through learning the response demands of the task (Hawkey et al. 2004). As noted earlier, the speakers in both the prior exposure and subsequent outcome conditions were different. Perceptual learning studies generally have shown that the idiosyncrasy of speakers is a key factor in the generalization from voice learning to the linguistic processing of speech tokens (e.g., Nygaard et al. 1994; Nygaard & Pisoni 1998; Bradlow et al. 1999). Cross-sensory studies also have shown that the familiarity with a speaker in one modality (A or V) is a key factor in subsequent gain in another modality (V or A; Rosenblum et al. 2007; Sanchez et al. 2013). Note that in our prior studies, the AV facilitation effect occurred in the conditions where the speakers were different in the prior exposure and in subsequent outcome conditions. Moreover, the speech materials were also different (consonants and words in the prior exposure versus the HINT in the subsequent outcome condition). Because of these differences, we argue that perceptual priming, perceptual learning, and procedural learning cannot alone be responsible for the larger gain provided by prior AV speech exposure compared with prior A speech exposure.

Further, we reason that the perceptual doping hypothesis differs from the notion of AV recalibration (Bertelson et al. 2003). Bertelson et al. (2003) studied the McGurk effect (a combination of incongruent A /aba/ and V /aga/ results in the AV percept /ada/; McGurk & Macdonald 1976), in which participants were exposed to an ambiguous phoneme sound between /aba/ and /ada/ (A?) dubbed into the visual articulation of either /aba/ or /ada/ (A?Vb or A?Vd). In a subsequent A test condition, the subsequent ambiguous A sound halfway between /b/ and /d/ was frequently perceived as /d/ following exposure to A?Vd or /b/ following exposure to A?Vb. That is, prior exposure to each of those incongruent AV speech tokens subsequently increased the proportion of responses corresponding to the V stimulus in the following A task. Bertelson et al. referred to this perceptual bias as “cross-modal (or phonetic) recalibration.” They suggested that prior AV exposure shifts (or adapts) subsequent A perception of those speech items, in favor of their V components. In addition, no such phonetic recalibration effect was observed if the participants were exposed to *nonambiguous and congruent* AV tokens (AdVd and AbVb). Bertelson et al. argued that the absence of such a recalibration effect in the nonambiguous and congruent AV conditions is due to a lack of intersensory conflict between the A and V speech signals that generates phonetic recalibration. Subsequent studies have also shown that cross-modal recalibration is speaker and token specific (Reinisch et al. 2014; van der Zande et al. 2014), as the phonetic recalibration is more evident if subsequent A items are the same as prior AV tokens, and the speakers are the same in both in prior and subsequent conditions.

However, the perceptual doping idea does not mean that subsequent A processing should necessarily be adapted to the V component of prior AV speech materials. Based on our prior findings, in fact, prior exposure to *congruent and degraded* (by background noise or in people with hearing loss) AV speech stimuli recalibrates phonological and lexical maps in the mental lexicon, which in turn facilitates the processing of subsequent A degraded speech signal for identification. As noted earlier, the perceptual doping is independent of the idiosyncrasy of speakers during speech stimuli exposure and in subsequent conditions. In terms of methodology, our past and present studies are different from phonetic recalibration studies, as the latter used incongruent AV tokens, and the A component of those incongruent AV tokens artificially became ambiguous in the continuum of /ba–da/.

Although we did not investigate the effect of prior AV speech stimuli on subsequent V speech processing, we speculate that early AV speech exposure would subsequently facilitate V speech processing (although examination of this effect is beyond of the scope of this study). This notion is based on an amodal account of speech perception (common to all sensory modalities, see the review by Rosenblum 2008). According to this theory, the specific modality of the input signal is irrelevant in the processing of a speech signal, from the very beginning when the V and A cues of an input signal are extracted and mapped with an amodal phonological or lexical representation. This notion is supported by studies showing that, at the phonological level, prior AV speech training resulted in both better A and V consonant identification (Hazan et al. 2005; Shinohara, Reference Note 2). At the lexical level, we could not find any study that investigated the effects of AV speech training on V word recognition. Based on the existence of a common recognition system underlying the V and A lexical recognition system (Auer 2002; Feld & Sommers 2011), one may argue that the recalibrated lexical maps associated with prior AV speech stimuli will lead to improved V word recognition following AV speech exposure, as the lexical maps become more distinctive and easily accessible.

The perceptual doping hypothesis was based on the findings of neuroimaging studies investigating the temporal mechanisms associated with AV speech correlates (Zion Golumbic et al. 2013; Crosse et al. 2015). For instance, Crosse et al. (2015) revealed that congruent AV speech presentation enhanced cortical representation of the speech envelope in a noise-free condition. Zion Golumbic et al. (2013) showed that viewing a speaker’s face enhanced the capacity of the auditory cortex to track the temporal speech envelope of that speaker. In a lexical access study, Li et al. (2011) showed that AV over A presentation facilitated neural semantic access, either in terms of within-class reproducibility (discriminability of semantic content within the same semantic category) or between-class discriminability (discriminability of semantic content between two different semantic categories). In addition, a recent animal study by Atilgan et al. (2018) showed that the association of V cues with an A stimulus enhanced spiking neural representations of that A stimulus in the auditory cortex.

Shams et al. (2011), in their review, purposed that prior multisensory exposure subsequently improves unisensory processing of stimuli, probably because prior multisensory experiences promptly create new connections between unisensory cortical areas in the brain. Shams et al. also suggested that prior multisensory experiences may recalibrate unisensory maps or unisensory representations of stimuli (i.e., V or A representations of stimuli) in a multisensory way.

The present study aimed to further explore the perceptual doping hypothesis using speech data obtained as part of the n200 project (see Rönnberg et al. 2016, for detailed information about the n200 project). In short, the n200 project is an ongoing longitudinal research project focusing on the interaction between speech signals and cognition in aided listeners with hearing loss. The “n200” refers to the sample of participants in this project (n = 200).

Among the broad variety of physiological, speech, and cognitive tests used in the n200 project, there were four speech tasks for which data were collected in both A and AV modalities. These four speech tasks are as follows. The first two tasks were gated identification of phonemes, where the participants were presented with successive fragments of either consonants or vowels, and their task was to guess a consonant or vowel that can be a continuation of the presented fragment(s). The third task was vowel duration discrimination, where the participants judged the duration of two vowels that varied in terms of duration. The fourth and final task was sentences in noise (Samuelsson & Rönnberg 1993), where the participants identified sentences in noise, with or without prior semantic context.

In all of these speech tasks, the order of modality (AV and A) presentation for the speech tasks was counterbalanced across participants, such that half of the participants started with the AV modality and the other half started with the A modality. Of note, counterbalancing is a common method used in experimental studies to control for order effects in repeated measures designs; it involves randomly determining the order of conditions for each participant. This type of counterbalancing has been used in prior speech perception research in which different speech stimuli modalities (AV, A, and V) were presented to participants in studies with a within-subjects design (e.g., MacLeod & Summerfield 1987; Grant et al. 1998; Sommers et al. 2005; Tye-Murray et al. 2007; Jesse & Janse 2012).

In fact, the present study attempted to evaluate the perceptual doping hypothesis by investigating the effects of modality order (or counterbalancing) in some of the speech tests administered in both A and AV modalities in the n200 study. Based on the perceptual doping hypothesis, we predicted significant differences in the A performance of speech stimuli between the two different modality orders. We posited that participants’ A performance in the AV1–A2 modality order would be better than their A performance in the A1–AV2 modality order, in each of the three speech tasks, because of prior AV speech processing (even if only of a short duration, e.g., around 5 min; see Materials and Methods). With regard to this short AV exposure in the present study, Wozny and Shams (2011) reported that even very brief prior exposure to asynchronous AV stimuli (a few milliseconds exposure) subsequently recalibrated the participants’ performance in an A spatial location task. We also predicted that prior A exposure would have a smaller effect than prior AV exposure on the subsequent AV processing of speech stimuli, based on our prior studies. Consequently, we assumed that the differences in the identification of AV speech stimuli between the two modality orders would not be statistically significant.

We should acknowledge here that a better setup to evaluate the perceptual doping hypothesis would involve a comparison between four orders of modality presentation, namely A1 and A2; AV1 and A2; AV1 and AV2; and A1 and A2. Nevertheless, the motivation of this article arose from our prior research. Specifically, we expect that the present article will provide a basis for future researchers to evaluate hypotheses or generate new models concerning an AV speech facilitation effect.

## MATERIALS AND METHODS

### Participants

A detailed description of the participants in the n200 project is available in the article by Rönnberg et al. (2016). In brief, 200 native Swedish listeners with hearing loss (114 males and 86 females), with bilateral, symmetrical, mild-to-severe sensorineural hearing loss, took part in this project. The participants were selected randomly from a list of patients at Linköping University Hospital, Sweden, who had given written consent to participate in this project. The Linköping regional ethical review board approved the project (Dnr: 55-09 T122-09).

The mean age of the participants was 60.95 years (SD = 8.42, range = 33–80 years). All the participants were habitual hearing aid users who had used their hearing aid for at least 1 year at the time of testing. The mean hearing threshold (without hearing aids) across seven frequencies (250, 500, 1000, 2000, 4000, 6000, and 8000 Hz) was 43.68 dB HL (SD = 10.27) for the right ear and 43.57 dB HL (SD = 9.92) for the left ear.

The participants reported themselves to be in good health, with no history of neurological disorders (e.g., Parkinson disease, stroke). The participants had normal or corrected-to-normal vision with eyeglasses.

In the n200 project, one participant did not complete the gated phoneme identification task, one did not complete the vowel duration discrimination task, and one did not complete the sentence identification in noise task, leaving data from 199 participants for the analysis of the speech tasks in the present article. Of note, the participants performed the above speech tasks using an experimental hearing aid (for more information about the type of amplification and the delivery of amplified speech to the participants, see Linear Amplification and Procedure sections below).

### Speech Stimuli

#### Gated Phoneme Identification Task

A description of the gated phoneme identification task is available in Moradi et al. (2017a). That study reported the extent to which the addition of V cues differentially contributed to the AV identification of consonants and vowels (using participants in the n200 project) in terms of improving recognition and reducing cognitive demands.

##### Consonants

Five Swedish consonants, structured in a vowel-consonant-vowel format (/ala, afa, ama, ata, asa/), were presented to the participants in both an A and an AV modality. The first vowel (/a/) was presented in full, and the gating started immediately at the onset of the consonant. The gate size was 40 ms: the first gate included the vowel (/a/) plus the initial 40 ms of a given consonant. The second gate added a further 40 ms of the consonant (a total of 80 ms of the consonant), and so on. The dependent variable in the present study was the mean IP (as defined in the Introduction) for consonants. The consonant gating task took about 7 min to complete.

##### Vowels

Five Swedish vowels, structured in a consonant-vowel format (/ma: my vi pI ma/), were presented to the participants in both an A and an AV modality. The selected vowels varied in terms of duration (/a: i:/ were the long vowels and /I a Y/ were the short vowels) and mouth shape (/i: I Y/ and /a/). This consonant-vowel format was used because previous studies revealed that when vowels are presented in consonant-vowel-consonant format, the critical acoustic and articulatory features of target vowels are not always distinguishable (Lindblom 1963; Stevens & House 1963). The consonant-vowel format was chosen to deliver better acoustic cues and clear articulation of a given vowel to listeners with hearing loss. The gate size was 40 ms, as in the consonant gating task. The dependent variable in the present study was the mean IP for vowels. The vowel gating task took around 7 min to complete.

A video camera was used for the video recordings of phonemes. The consonants and vowels were read by a male native Swedish speaker with natural articulation while looking into the camera. The video frame rate of recordings was 25 frames per second, with a resolution of 720 × 576 pixels. The face, hair, and upper part of the speaker’s shoulders were visible. An electret condenser microphone attached to the camera recorded the A phonemes at a sampling rate of 48 kHz, and the bit depth was 16 bits. The recorded phonemes were saved as “.mov” files and then edited into short clips (gates) to be played in the gating format.

#### Vowel Duration Discrimination Task

In Swedish, vowel duration is an acoustic feature that plays a critical role in separating words from each other. For instance, depending on whether /a/ is pronounced short /a:/ or long /a/, /“hal”/ either means “slippery” with a long vowel or “hallway” with a short vowel. The vowel duration discrimination task used in the present study was initially developed by Lidestam (2009). In that task, the participants were exposed to two different syllables in a consonant-vowel-consonant format (/lal/ or /mam/) in which vowel duration (/a/) was varied. The task was presented to the participants in both A and AV modalities. The participant’s task was to report which syllable (the first or second syllable presented) was longer. Of note, the two consonant contexts differ in terms of V saliency, as /m/ is more visually distinct than /l/ (e.g., /l/ is pronounced inside the mouth and without lip closure, while /m/ is pronounced by lip closure).

In the study by Lidestam (2009), each video file of /mam/ and /lal/ was edited into 13 separate speech tokens; the number of frames in each file varied. Each frame contained 33 ms duration of vowel /a/. For instance, the longest file of /mam/ had the maximum number of 13 frames (file number 13). Another file of /mam/ had 1 frame fewer than the longest file (file number 12), and another had 1 frame fewer (file number 11); file number 1 had 12 frames fewer than file number 13. In the shortest speech tokens, frames were removed from the middle of the sequence, with vowel /a/ toward the beginning and the end of the vowel. In total, there were 156 (13 × 12) vowel duration discrimination tokens in each context of /mam/ and /lal/; the participants had to distinguish two separate clips from each other.

In the n200 project, only 5 of the 13 available clips were selected from the task used by Lidestam (2009) in each context of /mam/ and /lal/. All five clip durations were compared against all other tokens (5 × 4); hence, there were 20 pairs of tokens in each context. Thus, there were four pairs where the first token was longer than the second token by 1 step (defined as 33 ms), four pairs where the second token was longer than the first token by 1 step, three pairs where the first token was longer than the second token by 2 steps (or 66 ms), three pairs where the second token was longer than the first token by 2 steps, and so on, down to only one pair with the maximum difference of +4 steps and one pair with the maximum difference of −4 steps. The pairs of tokens were quasi-randomized into one presentation order. No more than three consecutive presentations had a longer first (or second) token; no more than three consecutive presentations were of /lal/ or /mam/ tokens. In addition, step difference (−4 to 4) and speech token duration (the different lengths of token pairs, ranging from shortest-shortest to longest-longest) were distributed across the list. In total, 40 test items were presented in A and AV modalities. The dependent variable was the number of errors made. The task took approximately 7 min to complete.

Recordings of /mam/ and /lal/ were made, spoken by a native Swedish male. A laptop was used to record and edit the video and audio files of syllables. The frame rate of video recordings was 29.97 frames per second, with a resolution of 640 × 480 pixels. The sampling rate of the recordings was 44.1 kHz, and the bit depth was 16 bits. The audio files were then exported from the video files and edited in the same fashion with regard to the sequences that were removed. The audio and video files were then merged, creating new files.

Similar to Lidestam (2009), we report the number of errors, and not the percentage of correct answers. The results would not be qualitatively different if we reported correct responses or errors; indeed, the test statistics, such as means, variability, and group sizes, would be identical. For the purposes of the present study, we only report the extent to which modality order affected vowel discrimination ability in general; errors were pooled in the two contexts of /mam/ and /lal/. Future studies will report on the extent to which V cues affect vowel discrimination ability in persons with hearing loss and examine the relationships between vowel duration discrimination ability and other speech, cognitive, and physiological variables.

#### Sentences-in-Noise Identification Task

This task was designed by Samuelsson and Rönnberg (1993) to study the effects of prior script cues on sentence-based lipreading. The task comprises three different script cues about specific events that occur within those script cues: a clothing store, a train, and a restaurant. Within each context, there are two types of sentences: typical sentences (e.g., “can we pay for our dinner by credit card”) and atypical sentences (e.g., “can you hang my overcoat beside the dark coat?”).

The sentences-in-noise speech materials were recorded specifically for use in the n200 project (interlaced). In the n200 project, the sentences-in-noise task consisted of 48 sentences presented in A and AV modalities. For each modality type, there were 24 sentences, consisting of 12 sentences with prior script cues (e.g., a clothing store, a train, and a restaurant) and 12 sentences without those prior script cues. Within each set of 12 sentences, there were six typical sentences and six atypical sentences.

A speech-shaped noise was added to the presentation of sentences to avoid a ceiling effect in performance. To generate the background noise, the root mean square (RMS) of each sentence waveform was computed and the sentences were subsequently rescaled to the same RMS level. Then, the average long-term spectrum of the sentences was computed, and a random noise with the same spectral properties as the speech signal was used as the background noise. Specifically, a 128-coefficient finite impulse response filter was plotted to correspond to the long-term spectrum of sentences. White noise was filtered via the above filter and scaled to the equivalent RMS amplitude as the sentences.

The signal to noise ratio (SNR) was adjusted individually for each participant on the basis of his or her HINT (Hällgren et al. 2006) score at a 50% correct level; the SNR for an individual participant was set at −1 dB SNR below his or her HINT score at the 50% correct level. The HINT is a sentences-in-noise identification task that consists of daily sentences comprising three to seven words on a background of steady state speech-shaped noise. Participants were first familiarized with a 10-sentence practice list. To determine the SNR for each participant, a 20-sentence experimental list was used. The first sentence in both the practice and experimental lists was presented at 65 dB SPL and 0 dB SNR. The participants were asked to listen and repeat each sentence. An automatic, adaptive up-down procedure was used to determine the SNR of each participant at a correct response rate of 50%. If all words were correctly repeated, the SNR was decreased by 2 dB, and if one or more words were not correctly repeated, the SNR was raised by 2 dB. The HINT took around 10 min to complete.

The video frame rate of recordings in the Samuelsson and Rönnberg sentences-in-noise task was 25 frames per second, with a resolution of 720 × 576 pixels. An electret condenser microphone attached to the camera recorded the A stimuli at a sampling rate of 44.1 kHz, with a bit depth of 16 bits. The recorded stimuli were saved as “.avi” files. The sentences-in-noise task took around 15 min to complete.

For the purposes of the present study (similar to the vowel duration discrimination task), we only report the extent to which the modality order affected sentence identification in noise in general. The extent to which typicality (atypical versus typical sentences) and modality (AV versus A) affect sentence identification in noise will be reported in future studies.

In the n200 project, all data were collected at three separate sessions; each session took between 2 and 3 hr to complete. The gating task, vowel duration discrimination task, and the sentences-in-noise task were carried out in session three, along with the other speech tasks. The order of speech data collection in session three was as follows: the Swedish HINT (a test of A sentence-in-noise identification), the Samuelsson and Rönnberg sentences-in-noise task, the A inference-making test (a test of inference-making ability), the Hagerman sentence test (a test of A sentence-in-noise identification), the gated phoneme tasks, and the vowel duration discrimination task.

### Linear Amplification

Speech stimuli were linearly amplified for each participant, in order to assure audibility, using a voice aligned compression (VAC) rationale (Buus & Florentine 2002; see Ng et al. 2013, for technical details). VAC, an Oticon processing procedure, provides a linear gain at a 1:1 compression ratio to pure-tone input levels ranging from 30 to 90 dB SPL. The aim of VAC is to deliver greater compression at low input levels and less compression at high input levels through a lower compression knee point (i.e., increasing gain for weaker inputs). In other words, VAC aims to improve subjective sound quality. There was no background noise in the gated phoneme and vowel duration discrimination tasks. As mentioned earlier, a speech-shaped noise was added to the amplified speech signal in the sentences-in-noise task.

### Procedure

Participants were presented with speech stimuli while seated in a sound booth at Linköping University Hospital. For the gated phonemes and vowel duration discrimination tasks, a laptop equipped with Tcl/Tk and Quick TimeTel software was used to deliver the speech stimuli, collect responses, and monitor participants’ progress. For the Samuelsson and Rönnberg sentences-in-noise task, a desktop computer was used to present the speech stimuli. The laptop and desktop computer were located outside the sound booth and were configured for dual-screen presentation. A 17-inch Flatron monitor (LG L1730SF), positioned inside the sound booth, was used for V presentation of the speech stimuli. The V speech stimuli were viewed from a distance of about 50 cm. The monitor was turned off during the A presentation of the speech stimuli.

In order to transmit the amplified A speech signal to each participant, the laptop and desktop computer were routed to the input of an experimental hearing aid (Oticon Epoq XW, behind-the-ear type), placed in an anechoic chamber (Brüel & Kjær, type 4232). The output of the hearing aid was coupled with an IEC-711 ear simulator (Brüel & Kjær, type 4157). The A speech signal was then delivered via an equalizer (Behringer, Ultra-Curve Pro, model DEQ2496) and another measuring amplifier (Brüel & Kjær, type 2636) into a pair of ER3A insert earphones, inside the sound chamber, where the participants received the amplified speech signal.

A microphone in the sound chamber (routed into an audiometry device) transmitted the verbal responses of participants to the experimenter outside the sound booth. In the gated phonemes (consonants and vowels) and vowel duration discrimination tasks, participants responded orally and the experimenter wrote down the responses. In the HINT, the participants responded verbally, stating the sentences heard in noise, and the experimenter monitored whether each sentence had been correctly repeated on a desktop computer. In the Samuelsson and Rönnberg sentences-in-noise identification task, the participants again responded verbally and the experimenter wrote down the number of accurately repeated words in each sentence.

In the gated phoneme identification task, the participants began with consonant identification, followed by vowel identification. The presentation modality (A versus AV) within each gated phoneme task (vowels and consonants) was counterbalanced across participants: half of the participants started with the A identification of phonemes (both consonants and vowels) and the other half started with the AV identification of phonemes (both consonants and vowels). The participants received written and oral instructions on how to perform the gated phoneme identification task. The experimenter encouraged the participants to attempt identification after each gate of presentation of a given phoneme, irrespective of how unsure they were about the correctness of their response. The experimenter gave no feedback with regard to correctness or incorrectness of responses during the presentation of gated stimuli. If a participant correctly identified a phoneme at a given gate, the presentation of gates proceeded until three continuous correct responses had been given, to avoid random guessing. Three consecutive correct responses were considered as a correct response, and the IP recorded was the first gate at which the participant gave a correct response. The presentation of gates for that speech token was then stopped and the experimenter started the gating for the new token. When an item was not correctly identified, its entire duration plus one more gate size was calculated as the IP for that token. This scoring procedure corresponds to that of previous studies that have used the gating paradigm (Elliott et al. 1987; Metsala 1997; Moradi et al. 2013, 2014b; Lidestam et al. 2014).

In the vowel duration discrimination task, the participants were informed that the duration differences were meant to be difficult to detect in many cases but that they should do their best. They pressed buttons to indicate whether the first speech token (red button, on the left side on the table in front of them) or the second token (green button, on the right side) was longer. The participants began with /mam/, followed by /lal/. The modality type (AV versus A) within each context (/mam/ and /lal/) was counterbalanced across participants, such that half of the participants began with the A modality (in both /mam/ and /lal/ tokens) and the other half started with the AV modality (in both /mam/ and /lal/ tokens). Missing responses were scored as errors. The participants had some practice at the task (six examples of increasing difficulty) to become familiar with the procedure before the real experimental trial began.

For the gated phoneme and vowel duration discrimination tasks, there was a 5 sec time-out; if no response was given within 5 sec in each trial, the participant proceeded to the next trial.

For the sentences-in-noise task, the participants were asked to repeat what they heard. The modality type (AV and A), semantic context types (clothing store, a train, and a restaurant), and sentence typicality (atypical and typical) were counterbalanced within participants. Half of the participants began the task with the AV modality and the other half with the A modality. The task was scored in terms of the number of words correctly repeated across whole sentences in the task. The task was scored on a word-by-word basis. Partially correct words or similar sounding words were considered as incorrect.

Note that in all the abovementioned speech tasks, the modality orders (e.g., A1–AV2, or AV1–A2) were fixed for each participant. For instance, if a participant started with AV presentation and then received A presentation for the sentences-in-noise task, he or she started with AV presentation and then received A presentation for the vowel duration discrimination and the gated phonemes tasks too.

### Analysis

For the analysis, we used a 2 × 2 Modality Order (first and second) × Modality Type (A and AV) analysis of variance (ANOVA), with repeated measures on the second factor in each speech task. We also performed planned comparisons, using unpaired *t* tests to assess the differences between A1 and A2, and between AV1 and AV2, to determine whether A and AV speech scores were significantly different from each other between the two modality orders for each modality type. In addition, we utilized paired *t* tests to evaluate the extent to which adding V speech cues facilitated the identification of speech stimuli in each modality order (AV1 versus A2 and AV2 versus A1).

Perceptual doping is indicated if there are main effects of modality order (i.e., if AV1–A2 is better than A1–AV2), if there are interactions between modality order and type, or a combination of main and interaction effects, and under the corollary condition that *the difference between A1 and A2 is greater than the difference between AV1 and AV2*.

## RESULTS

### AV Speech Facilitation in a Gated Consonant Identification Task

**Fig. 1.**
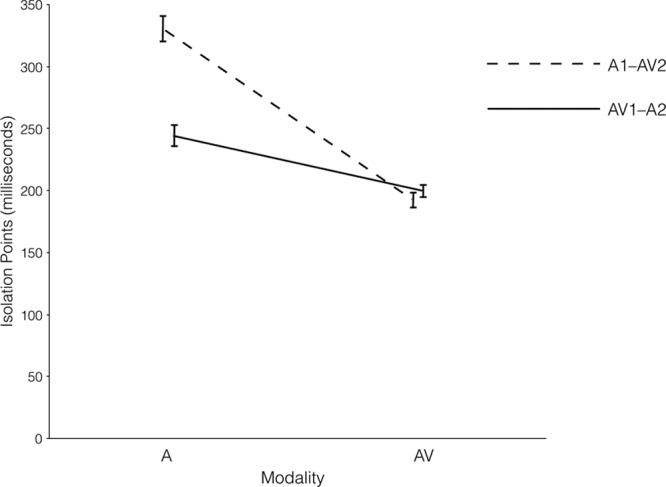
Audiovisual (AV) and auditory (A) isolation points (IPs) of consonants in modality orders of AV1–A2 and A1–AV2.

Figure 1 displays the mean IPs for consonants in the A and AV modalities, according to the modality orders. A Modality Order × Modality Type ANOVA for consonants showed significant main effects of modality order (*F* (1, 197) = 18.82, *p* < 0.001, 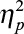 = 0.09) and modality type (*F* (1, 197) = 243.56, *p* < 0.001, 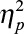 = 0.55). In addition, the interaction between modality order and modality type was significant (*F* (1, 197) = 63.37, *p* < 0.001, 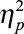 = 0.24). This interaction suggests that the effect of modality order (A1–AV2 versus AV1–A2) on the IPs of consonants is dependent on the modality type of consonants (A versus AV).

The perceptual doping hypothesis suggests that a relatively larger gain is achieved by prior AV speech exposure than prior A exposure for the subsequent identification of consonants. Consequently, based on our a priori hypothesis, we expected a significant difference in A IPs between the modality orders of AV1–A2 and A1–AV2. In addition, we expected that the difference in AV IPs between those modality orders would not reach statistical significance because of the smaller gain provided by the prior A exposure.

In order to evaluate this, planned comparisons were conducted to examine the differences between mean A and AV IPs of consonants for different modality orders. The results showed a significant difference in the A identification of consonants between the two modality orders, as the mean A IP in the AV1–A2 modality order was significantly shorter (representing faster identification) than that in the A1–AV2 modality order (*t* (197) = 6.54, *p* < 0.001, *d* = 0.93). There was no significant difference between the different modality orders in terms of AV IPs (*t* (197) = 0.97, *p* = 0.332; see Fig. 1).

In addition, we were interested in evaluating the extent to which AV over A presentation would impact on the IPs of consonants in each modality order. The results showed that the AV relative to A presentation resulted in shortened IPs for consonants in both modality orders of A1–AV2 (*t* (99) = 15.53, *p* < 0.001, *d* = 1.55) and AV1–A2 (*t* (98) = 5.88, *p* < 0.001, *d* = 0.59).

### AV Speech Facilitation in Gated Vowel Identification Task

**Fig. 2.**
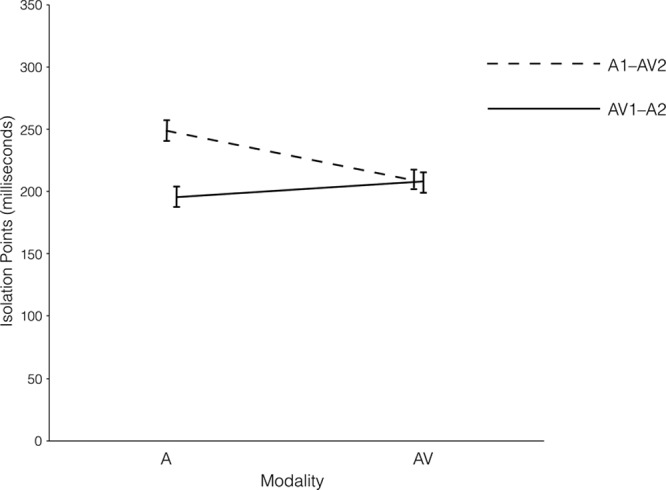
Audiovisual (AV) and auditory (A) isolation points (IPs) of vowels in modality orders of AV1–A2 and A1–AV2.

Figure 2 shows the mean IPs for vowels in the A and AV modalities, according to the modality orders. A 2 × 2 Modality Order × Modality Type ANOVA for vowels showed significant main effects of modality order (*F* (1, 197) = 6.91, *p* = 0.009, 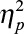 = 0.03) and modality type (*F* (1, 197) = 7.54, *p* = 0.001, 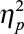 = 0.04), together with an interaction effect (*F* (1, 197) = 27.25, *p* < 0.001, 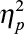 = 0.12).

Planned comparisons revealed a significant difference in mean A IPs for vowels between the two modality orders, as the mean A IP in the AV1–A2 modality order was significantly shorter than that in the A1–AV2 modality order (*t* (197) = 4.62, *p* < 0.001, *d* = 0.66). There was no significant difference between the different modality orders in terms of AV IPs (*t* (197) = 0.13, *p* = 0.900).

When comparing the IPs of AV over A presentation in each modality order for vowels, the results showed that, only in the A1–AV2 modality order, AV relative to A presentation resulted in shortened IPs (*t* (99) = 5.87, *p* < 0.001, *d* = 0.59). In the AV1–A2 modality order, however, the AV over A presentation yielded a nonsignificant difference (with a marginal advantage for A over AV presentation) in the IPs for the identification of vowels (*t* (98) = 1.68, *p* = 0.095).

### AV Speech Facilitation in the Vowel Duration Discrimination Task

**Fig. 3.**
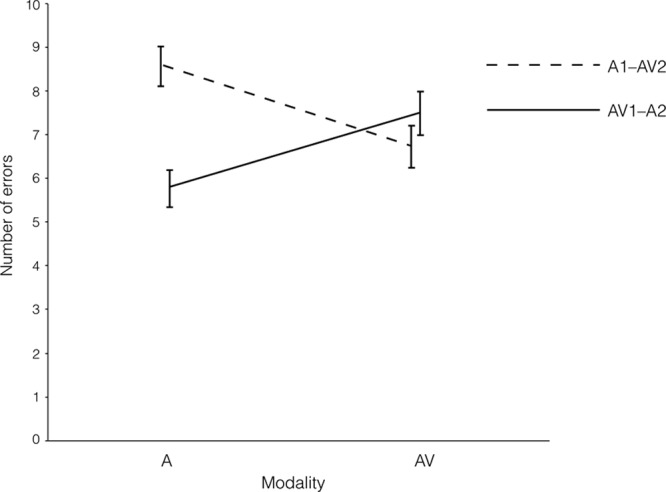
Audiovisual (AV) and auditory (A) number of errors in modality orders of AV1–A2 and A1–AV2.

Figure 3 displays the mean errors in the vowel duration discrimination task for the A and AV modalities, according to the modality order. A 2 × 2 Modality Order × Modality Type ANOVA on vowel duration discrimination ability showed no significant main effects (modality order, *F* (1, 197) = 3.06, *p* = 0.082; modality type, *F* (1, 197) = 0.05, *p* = 0.830. However, the interaction effect was significant (*F* (1, 197) = 32.45, *p* < 0.001, 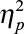 = 0.14). Although the main effects were not significant, the significant interaction effect indicates that the effect of modality order on vowel duration discrimination task performance was dependent on modality type.

Planned comparisons showed that the mean A error in the AV1–A2 modality order was significantly lower than that in the A1–AV2 modality order (*t* (197) = 4.45, *p* < 0.001, *d* = 0.64). Similar to the gated phoneme identification tasks, there was no significant difference between the different modality orders in terms of AV errors (*t* (197) = 1.05, *p* = 0.295).

When comparing AV over A presentation in terms of the number of errors made in each modality order, the results showed that the association of V cues with A presentation significantly reduced the number of errors in discriminating vowel duration in the A1–AV2 modality order (*t* (99) = 4.29, *p* < 0.001, *d* = 0.43). In contrast, in the AV1–A2 modality order, participants’ performance in the A modality was better than that in the AV modality (*t* (98) = −3.88, *p* < 0.001, *d* = 0.39; a negative contribution of V cues).

### AV Speech Facilitation in the Sentences-in-Noise Task

**Fig. 4.**
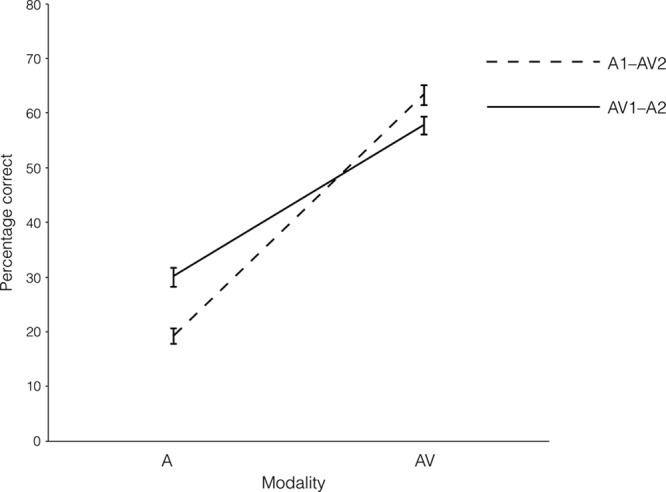
Audiovisual (AV) and auditory (A) percentage of correctly identifying words in sentences in background noise in modality orders of AV1–A2 and A1–AV2.

Figure 4 displays the mean number of correctly identified words in sentences presented in background noise (assessed using the sentences-in-noise task). A 2 × 2 Modality Order × Modality Type ANOVA on the correct identification of words in sentences presented in background noise was performed. The results showed a significant main effect of modality type (*F* (1, 197) = 1299.65, *p* < 0.001, 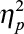 = 0.8) and an interaction effect (*F* (1, 197) = 66.22, *p* < 0.001, 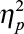 = 0.25), but the main effect of modality order was not significant (*F* (1, 197) = 1.45, *p* = 0.231).

Planned comparisons showed that the mean A identification of words in the AV1–A2 modality order was significantly higher than that in the A1–AV2 modality order (*t* (197) = 4.81, *p* < 0.001, *d* = 0.69). In contrast to the gated phonemes and vowel duration discrimination tasks, however, AV1 and AV2 were significantly different. The mean AV identification of words in the A1–AV2 modality order was significantly higher than that in the AV1–A2 modality order (*t* (197) = 2.29, *p* = 0.023, *d* = 0.32; see Fig. 4).

In addition, when comparing AV relative to A presentation for correctly identified words in sentences (presented in background noise), the results showed that AV over A presentation resulted in better performance in the modality orders of A1–AV2 (*t* (99) = 29.62, *p* < 0.001, *d* = 2.96) and AV1–A2 (*t* (98) = 20.98, *p* < 0.001, *d* = 2.11).

The *t* test results of multiple comparisons for all speech tasks used in the present study are summarized in Appendices A and B (supplementary material, Supplemental Digital Content 1, http://links.lww.com/EANDH/A449). Appendix A shows the *t* test results for the perceptual doping hypothesis evaluation (comparisons between AV1–A2 and A1–AV2). Appendix B presents the effects of AV over A presentation on the processing of speech stimuli for each modality order.

## DISCUSSION

The findings of the present study support the perceptual doping hypothesis by showing a relatively larger gain for prior AV speech exposure than prior A speech exposure for the subsequent processing of speech stimuli. In addition, the sentence identification in noise task findings was interesting; irrespective of the first modality presented (A1 or AV1), the participants performed better in the second modality presented (A2 or AV2). We hypothesize that, given the complexity of the speech materials in the sentences-in-noise task, a procedural learning effect, or a combination of *procedural learning and perceptual learning*, occurred after task completion in the first modality. This may have subsequently facilitated the identification of speech stimuli in the second modality presented (see below for a detailed discussion of perceptual doping and the procedural learning effect, and the possible additional perceptual learning effect, in the sentences-in-noise task). Nevertheless, the effect size of the perceptual doping effect was about twice the effect size of the procedural learning effect (or the combination of procedural learning and perceptual learning effects).

### Perceptual Doping in Gated Phoneme Tasks

The findings of the gated identification tasks support the perceptual doping hypothesis (see Figs. 1, 2). The means A IPs of both consonants and vowels in the AV1–A2 modality order were shorter (indicating better performance) than those in the A1–AV2 modality order. In addition, there were no significant differences in the AV IPs of both consonants and vowels between different modality orders.

van Wassenhove et al. (2005) reported that AV over A presentation reduced the amplitude of electroencephalography N1 and P2 responses. According to Näätänen and Winkler (1999), the P2 auditory evoked potential is a speech-specific feature that is presumably linked to the processing of physical characteristics of a speech sound before its categorization. Hence, it can be reasoned that AV speech stimuli speed up the identification of speech stimuli. Crosse et al. (2015) and Zion Golumbic et al. (2013) showed that AV speech exposure enhanced the cortical representation of temporal speech cues. Accordingly, we speculate that prior AV speech exposure facilitated the subsequent extraction of critical acoustic features required for the identification of consonants (e.g., temporal cues such as speech envelopes, Van Tasell et al. 1987) and vowels (e.g., formant frequency, Lindblom & Studdert-Kennedy 1967). This may have resulted in shortened A IPs for consonants and vowels in the modality order of AV1–A2 relative to A1–AV2.

**Fig. 5.**
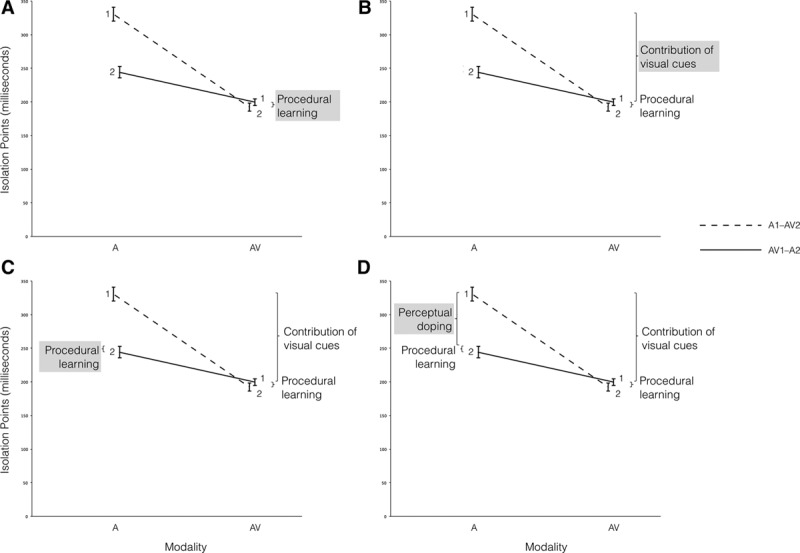
Audiovisual (AV) and auditory (A) isolation points (IPs) of consonants in modality orders of AV1–A2 and A1–AV2 explained in four steps.

We will now attempt to tease apart the theoretical components that we think underlie the data patterns (see Fig. 5A–D, taken from the gated consonant task, but the same principle applies to all four tasks). In this type of counterbalanced design, with modality types counterbalanced within subjects (A versus AV), and modality orders counterbalanced between subjects (first versus second task), there are several factors that can affect participants’ performance in the tasks. First of all, the lowest performance was established for the A gating in the A1–AV2 group (at A1). Those participants who had not been previously exposed to the task may have had some room for improving performance, as they had not yet learned to master the task. This procedural learning is the main explanation for the better performance of the A1–AV2 group at AV2 (see Fig. 5A). In Figure 5B, the difference between the A1–AV2 and the AV1–A2 groups for the AV presentation (i.e., A1 versus AV1) can be attributed purely to the contribution of V cues inherent in the AV presentation, as the effect of procedural learning has been subtracted (but is theoretically inherent in the A1–AV2 comparison). By assuming equal extensive procedural learning in the AV1–A2 and A1–AV2 modality orders, procedural learning only accounts for a small part of the gap between A1 and A2. The remainder of this gap is attributed to the so-called perceptual doping (see Fig. 5D). It should be noted that the above assumption of equal procedural learning between the two modality orders was made by the authors of this article; an accurate estimation of such procedural learning needs to be investigated by an alternative setup, such as by examining the differences between A1 and A2 or AV1–AV2 in a randomized control study.

### Perceptual Doping in the Vowel Duration Discrimination Task

Prior AV speech exposure provided a much larger gain than prior A exposure in terms of reducing the number of errors in a vowel duration discrimination task (see Fig. 3), which further supports the perceptual doping hypothesis. We speculate that prior AV exposure subsequently facilitated the extraction of durational cues (e.g., initial formant transitions, stress of syllables), which helped the listeners in the AV1–A2 group to auditorily discriminate vowel durations more accurately than the listeners in the A1–AV2 group. Interestingly, for the A1–AV2 modality order, the mean AV error in discriminating vowel duration was lower than the mean A error (see Fig. 3). This is in line with current research showing that the association of V cues with A speech stimuli improves the identification of vowels (e.g., Breeuwer & Plomp 1986; Moradi et al. 2017a). However, for the AV1–A2 modality order, the mean A error was lower than the mean AV error, suggesting a negative contribution of V cues to A speech stimuli in the discrimination of vowel duration. This negative visual effect is at odds with the findings of the A1–AV2 modality order and the current literature. Similarly, in the gated vowel identification task, AV relative to A presentation speeded up the identification of vowels for the A1–AV2 modality order, but no such effect was observed for the AV1–A2 modality order.

The perceptual doping hypothesis may help explain this odd finding. The perceptual doping effect in the AV1–A2 modality order may have been so strong that it greatly helped the listeners to decode the temporal cues necessary to discriminate vowel duration in the vowel duration discrimination task and to extract phonological cues for vowel identification in the gated vowel identification task, subsequently boosting the participants’ performance on these tasks in the A modality. The addition of V cues might have a stronger effect for consonants than vowels in terms of their AV identification (Kim et al. 2009; Moradi et al. 2017a), which could explain why the abovementioned effect for vowels was not observed in the gated consonant task. For instance, Moradi et al. (2017a) reported that the effect of adding V cues on AV identification and cognitive demand reduction was more evident for consonants than for vowels (i.e., more V saliency for the AV identification of consonants than vowels). Hence, it can be argued that in the modality order of A1–AV2, the less salient vowels’ V cues resulted in shorter IPs (in the gated vowel task) and fewer errors (in the vowel duration discrimination task). In the AV1–A2 modality order, however, the boosted A performance associated with prior AV exposure may have nullified the effects of less salient vowels’ V cues in AV identification and AV vowel duration discrimination compared with their counterparts in the A modality. Given the greater contribution of V cues in the AV identification of consonants, the difference between AV and A IPs was so large that it resulted in boosted A IPs that remained longer than the AV IPs in the AV1–A2 modality order.

One question that arises regarding the above argument is why the effect of perceptual doping in vowel duration discrimination ability in A2 was so much stronger than the effect of adding V cues on AV1 and AV2 conditions? One possible explanation might be a ceiling effect for the vowel duration discrimination task in the n200 study, as the task was conducted in silent listening conditions. The mean errors of participants for the A and AV modalities in this task were 7.17 and 7.09 (out of 20 items in each modality), respectively (see Table 2 in Rönnberg et al. 2016). In fact, the effect of adding V cues on AV speech perception has been reported to be more evident in noisy rather than silent listening conditions (e.g., Moradi et al. 2013). So, better AV speech performance in the AV1 and AV2 modality orders might be expected in the case of background noise in the vowel duration discrimination task, compared with A2 performance.

Further research on tasks performed in both silence and noise is required to investigate the extent to which the perceptual doping effect and AV presentation (over A presentation) affects a person’s ability to discriminate vowel duration and identify vowels and consonants. This can be done using between-subjects research experiments, comparing prior AV speech exposure with no such prior exposure (i.e., an A1–A2 and an AV1–AV2 group, respectively). By utilizing such a design, potential improvement in performance due to a change between modalities is eliminated, leaving only the effects of the contribution of V cues in silent and noisy conditions. We predict that there would be a larger main effect of modality (A or AV, between groups) and a smaller main effect of time (first versus second test, within groups), but no interaction between modality and time—which would indicate no perceptual doping.

### Perceptual Doping in the Sentences-in-Noise Task

The findings of this task are complex. Prior exposure to both AV and A speech stimuli resulted in significant subsequent improvement in A and AV sentence identification in noise, respectively. As mentioned above, we suggest that a procedural learning effect (due to the complexity of speech materials) or a combination of procedural learning and perceptual learning effects, in addition to the perceptual doping effect, subsequently facilitated participants’ performance in the second modality presented in the sentences-in-noise task. This argument is based on the findings of Wilson et al. (2003), who studied repeated and unique word recognition using sentence materials from the Veterans Administration Sentence Test (VAST; Bell & Wilson 2001), presented in silence, within five sessions over 5 to 10 days. Their findings showed a *procedural learning* effect associated with increasing familiarity with the test procedure (familiarity with the speaker, the listening response task, and the task environment). In their study, that learning was not representative of *content learning* (learning the word or sentence items). Similarly, Yund and Woods (2010) have reported small procedural learning effects when performing the HINT (Nilsson et al. 1994) using unique sentences during three sessions over a 10-day period.

We speculate that because the speech materials in the sentences-in-noise task were manifold (e.g., typical versus atypical, with and without semantic context), and varied in terms of context (at a restaurant, in a train, at a clothing store), a procedural learning effect occurred after the first modality was presented. That is, the participants learned the format of the task after the first modality (A1 or AV1) and that subsequently helped them respond to speech materials during the second modality (A2 or AV2). This procedural learning was presumably minimal in the gated phoneme identification and vowel duration discrimination tasks, as the speech materials in those tasks were less complex than those in the sentences-in-noise task. (There were only two different phonemes [consonants and vowels] in the gated tasks and two different contexts [/lal/ and /mam/] in the vowel duration discrimination task.)

We also speculate that a perceptual learning effect, in addition to a procedural learning effect, probably improved participants’ performance in the second modality (A2 or AV2) presented in the sentences-in-noise task. This speculation was, in fact, inspired by Davis et al. (2005), who showed a gradual improvement in correctly identifying words in distorted (noise vocoded) sentences during exposure to 30 distorted sentences. From our data, it is difficult to disentangle procedural learning from perceptual learning in order to estimate their contribution in the second modality order during the identification of words in sentences.

It would be interesting to study the extent to which procedural learning, perceptual learning, and perceptual doping influence A and AV sentence identification in different listening conditions (e.g., in silence or in background noise). This is important not only because the current literature on the interaction of procedural learning and perceptual learning in sentence identification is limited but also because little is known about the abovementioned perceptual facilitation effects in optimum and degraded listening conditions.

While Wilson et al. (2003) found procedural learning effects when using the VAST presented in silence, Yund and Woods (2010) found minimal procedural learning effects using the HINT. Yund and Woods argued that one explanation for this discrepancy might be the differences between the HINT and VAST sentence materials and the calculation of SNRs. So, it is not clear to what extent degraded speech materials enhance or decrease procedural learning in the process of correctly identifying words in sentences.

While little is known about perceptual learning for the identification of words in sentences in optimum listening conditions (e.g., in silence), studies of tasks performed in degraded listening conditions (e.g., noise-vocoded speech materials) have reported a perceptual learning facilitation effect on the identification of words in sentences (e.g., Davis et al. 2005; Huyck & Johnsrude 2012; Huyck et al. 2017). In fact, Huyck and Johnsrude (2012) revealed that attention to degraded sentences (or cognitive effort) is necessary for perceptual learning.

With regard to the perceptual doping effect, in the present study, we only used sentence materials with unique items, presented in background noise, for each modality order. The extent to which the type of listening conditions (e.g., clear versus distorted) would affect the perceptual doping effect on word identification in sentences requires further research. Moradi et al. (2017b) suggested that background noise (relative to a silent listening condition) may enhance the perceptual doping effect by focusing listeners’ attention (or cognitive effort) on the A and V components of a speech item, to form a coherent AV speech item from an incoming speech signal so as to map it to corresponding phonological or lexical representations. The data from gated consonants, gated vowels, and vowel duration discrimination tasks in the present study indicate a perceptual doping effect even in silence.

Assuming an equal degree of procedural learning (or procedural learning plus perceptual learning) in the two different orders of modality presentation, it can be argued that while there were only procedural learning effects in the A1–AV2 modality order, there were both perceptual doping and procedural learning effects in the AV1–A2 modality order, as the effect size in the latter was twice that of the former.

In summary, the results of the sentences-in-noise task suggest that although prior AV speech exposure facilitates subsequent A speech recognition, a procedural learning or procedural learning plus perceptual learning effect also facilitates the subsequent identification of speech stimuli, if the speech materials are manifold and complex and are presented in degraded listening conditions such as background noise.

To account for how perceptual doping occurs, Shams et al. (2011) suggested that early multisensory stimulation recalibrates unisensory maps, which results in more efficient unisensory processing of stimuli. Future research is needed to elucidate the neural signature of the perceptual doping effect.

### Perceptual Doping and Other Similar Perceptual Phenomena

Regarding the current study, we reason that the perceptual doping effect most probably differs from procedural learning and perceptual learning. For instance, if perceptual learning was applied to our results, one would expect a subsequent improvement in terms of the identification of AV stimuli after initial A speech exposure for the gated phonemes and vowel duration discrimination tasks too. However, participants’ identification of A speech stimuli in those tasks was improved only after prior AV speech exposure. Most importantly, the gated phoneme and vowel duration discrimination tasks results indicate a lack of perceptual learning or procedural learning in those speech tasks. We speculate that one probable explanation for the absence of perceptual learning or procedural learning in those speech tasks is a ceiling effect due to the presentation of those speech stimuli in silence (see below for a discussion on how the presentation of A and AV speech stimuli in silence may undermine a perceptual learning effect on the subsequent A processing of speech stimuli). Together, we agree with Shams et al. (2011) that an AV facilitation effect on subsequent unisensory processing involves different types of learning or perceptual mechanisms.

To interpret the present results in the context of perceptual learning, we hypothesize that early exposure to an AV speech signal (which is richer and more detailed in terms of acoustic components [e.g., place and manner of articulation] than early exposure to an A speech signal), which provides better feedback on the correct perceptual response, greatly helps listeners to better predict the identity of a speech item in a unisensory modality. Support for this interpretation comes from an A modality study by Lee et al. (2016), which showed that an acoustically rich signal (with high spectral details) versus a poor signal (with less spectral details) modulates the neural network involved in perceiving speech stimuli, with stronger activation in bilateral temporal, parietal, and frontal cortices.

So, it may be argued that an AV speech signal, which contains more rich and detailed acoustic cues than an A speech signal, results in greater activation of the neural networks involved in the perception of an A speech signal (see Zion Golumbic et al. 2013; Crosse et al. 2015). This consequently enhances the focus of participants’ attention on obtaining relevant cues for making a perceptual decision. This argument is in line with Bernstein et al. (2013), who demonstrated that A perceptual learning is enhanced by exposure to AV rather than A training materials. Bernstein et al. hypothesized that the availability of reliable V cues in an AV distorted speech signal, which per se is correlated with the A cues of that degraded speech signal, provides a top-down direction on the learning of novel A distorted speech tokens during AV training. In contrast, training with A degraded stimuli provides no extra information for the learning of particular cues (that may ease the identification of speech stimuli), apart from solely repeating the stimuli.

A recent study by Lüttke et al. (2016) showed that the recalibration effect which occurs after exposure to AV speech stimuli cannot be explained solely by perceptual priming or a learning effect, and there are other perceptual facilitation effects. These authors investigated A recalibration following the aforementioned McGurk effect (McGurk & Macdonald 1976). Lüttke et al. studied the extent to which experiencing the McGurk illusion influenced subsequent A phoneme identification in a forced-choice task using the three alternatives /aba/, /ada/, and /aga/. Promptly following the McGurk illusion, the A “aba” was more frequently perceived as “ada,” and this recalibration aftereffect was not due to perceptual priming or selective adaptation. Lüttke et al. proposed the existence of a phonetic recalibration following the McGurk illusion that shifts the phonetic maps of the “aba” signal onto an “ada” representation.

In sum, with our results, it is difficult to dissociate perceptual learning, enriched with prior AV exposure, from perceptual doping. More experimental evidence is needed to claim that the so-called perceptual doping effect is a special case of perceptual learning.

In three tasks in the present study (gated consonants, gated vowels, and vowel duration discrimination), there was almost no improvement following A exposure of speech stimuli, which is at odds with findings of A perceptual learning studies. The latter have shown that A comprehension improves with continued exposure to distorted speech, such as noise-vocoded speech (e.g., Davis et al. 2005; Hervais-Adelman et al. 2008). In fact, such A perceptual learning has also been observed in other adverse listening conditions, as when perceiving heavily accented speech (e.g., Clarke & Garrett 2004) or time-compressed speech (Peelle & Wingfield 2005). We reason that the lack of such A perceptual learning in the three aforementioned speech tasks was most likely due to nondistorted speech items in those speech tasks, which, to some extent, inhibit the occurrence of perceptual learning in those speech tasks. In the sentences-in-noise task, there was background noise and an improvement in comprehending sentences following each modality order. However, our design cannot disentangle procedural learning (due to the complexity of speech materials) from perceptual learning.

As noted in the Introduction, phonetic recalibration occurs when the A component of an incongruent AV speech signal is ambiguous (Bertelson et al. 2003). The current study demonstrated the effect of perceptual doping in the speech tasks presented in silence, as the A component of the congruent AV speech signal was not ambiguous. As noted in the Introduction, perceptual doping is independent of the idiosyncrasy of speakers; in addition, the occurrence of the perceptual doping effect denotes another difference between phonetic recalibration and perceptual doping when the A component of the prior congruent AV speech signal is unambiguous.

One point that should be discussed here is the recalibration both in the phonetic recalibration (Bertelson et al. 2003) and in perceptual doping. Phonetic recalibration refers to a readjustment of an existing phonetic representation that shifts the subsequent ambiguous A signal toward the V component of a prior incongruent AV speech signal (see Vroomen & Baart 2012). Recalibration in perceptual doping refers to the readjustment of an existing phonological or lexical representation following prior exposure to a congruent AV speech signal that eases the processing of a subsequent A speech signal in terms of its identification or detection. In other words, the subsequent readjustment in perceptual doping is not based on the V component of prior AV speech signal. Rather, it is based on updating unisensory perceptual processing following exposure to an AV speech signal (Shams et al. 2011).

We also hypothesize that perceptual doping is a nonconscious process (similar to perceptual priming or learning), as a listener is not consciously aware that what is going to be identified from A speech stimuli later on is eased by prior AV speech exposure.

As the speakers were the same in the above speech tasks, the findings of the present study are in line with the AV feed-forward model (Riedel et al. 2015), which tries to explain how brief initial AV exposure to familiar speakers improves subsequent A speech recognition of those speakers. According to the AV feed-forward model, the brain rapidly and effortlessly grasps the A and V characteristics of “a new speaker” and forms an AV simulation of that speaker. If the V signal is not available, the AV simulation feeds back to A brain areas and facilitates the voice (A) identification of that person. Note that in our previous articles (Moradi et al. 2013, 2017b; Lidestam et al. 2014), the speakers in the gated tasks and the HINT were different. Our previous research indicates that the perceptual doping effect is independent of speaker idiosyncrasy, as only one exposure to AV speech stimuli (with the same or different speakers in the initial exposure and following the A speech task) is sufficient to obtain subsequent improvement in A speech identification. Further research is needed to study the facilitatory effects of AV speech on subsequent A improvement when the speakers are the same or different.

It is important to disentangle the contribution of V cues on the AV facilitation effect upon subsequent A speech processing. In the present study, there were no V1 and V2 modality orders to compare with AV1–A2 and V1–A2 on subsequent A processing of speech stimuli. One question that arises is whether the effect of prior AV stimuli on subsequent A processing is solely due to the V component of the AV speech signal or whether a combination of congruent A and V speech stimuli is necessary. As noted in the Introduction, cross-modal studies have shown that prior lipreading subsequently improved the A identification of speech stimuli (Rosenblum et al. 2007; Wu et al. 2013). Rosenblum et al. (2007), however, reported that this V facilitation is a speakers’ idiosyncrasy effect, as the similarity of speakers in the training materials and following outcome measure is a key factor. Comparing AV versus V speech stimuli on subsequent A processing would be an interesting research topic, to scrutinize the role of V cues alone and in combination with an A signal on subsequent A processing.

### Perceptual Doping and Aural Rehabilitation of Listeners With Hearing Loss

Hearing aids are the most common means of aural rehabilitation in people with hearing loss. However, hearing aids cannot fully compensate for the speech recognition difficulties of listeners with hearing loss to the same degree as their normal-hearing counterparts (Dimitrijevic et al. 2004; Moradi et al. 2014a). Since the 1970s, different methods of A training have been devised to more fully compensate for the speech recognition difficulties in people with hearing loss (Bode & Oyer 1970; Rubinstein & Boothroyd 1987; for reviews see Sweetow & Palmer 2005; Henshaw & Ferguson 2013). However, the efficiency of those A training program has been reported to be low and not very robust (see Henshaw & Ferguson 2013; Ferguson et al. 2014). We predict that the association of congruent V speech cues with an A training program will boost their effects, even with shorter training sessions than those for A training, in terms of improving the listening capabilities of people with hearing loss (see Moradi et al. 2017b).

In terms of semantic and lexical rehabilitation, hearing loss has been associated with deleterious effects on lexical and semantic representations in the mental lexicon (Rönnberg et al. 2011; Classon et al. 2014). This adversely affects the mapping process between the speech signal and the corresponding lexical and semantic representations. Li et al. (2011) showed that AV speech exposure improves semantic access in normal-hearing listeners (by enabling the discrimination of semantic content, both within the same category and between different semantic categories). Hence, it can be hypothesized that AV speech training not only amplifies bottom-up processing (e.g., by enabling the extraction of phonological cues), which could aid the identification of speech stimuli, but also recalibrates the lexical and semantic maps in long-term memory, which may slow the degeneration of lexical and semantic representations in people with hearing loss.

The extent to which cognitive impairment affected the integration of A and V speech signals and the perceptual doping effect is unknown, and this is a limitation of the present study. Studies have shown that hearing loss is independently associated with cognitive impairment and Alzheimer’s disease (Lin et al. 2011; Gurgel et al. 2014; see Zheng et al. 2017 for a meta-analysis on the link between hearing loss and Alzheimer’s disease). In addition, studies have reported that cognitive impairment and Alzheimer’s disease can result in delayed AV integration ability (Wu et al. 2012) and a deficit in AV bottom-up integration ability (Festa et al. 2017). In the n200 study, only the Mini-Mental State Examination (MMSE; Folstein et al. 1975) was used to evaluate the general cognitive function of those with hearing loss, with no further medical evaluation. We suggest that future research investigating multisensory speech perception in people with hearing loss uses a precise evaluation of cognitive function in people with hearing loss, using the MMSE with further clinical evaluation, or the Montreal Cognitive Assessment (Nasreddine et al. 2005), which is reported to have better sensitivity in detecting mild cognitive impairment than the MMSE (see Nasreddine et al. 2005; Dong et al. 2012). In addition, the extent to which cognitive impairment or Alzheimer’s disease influences the perceptual doping effect would be an interesting research topic.

In the present study, the consonant context in the gated vowel task was varied across different vowels. As noted in the Method, the use of varied consonant contexts in the gated vowel task aimed to deliver clear acoustic and articulatory cues of each vowel to listeners with hearing loss; however, these contexts might have generated a learning effect in the second modality order presented. We recommend that future studies avoid these potential learning effects when studying vowel identification at separate time points in which each vowel has a specific consonantal context.

## CONCLUSIONS

The findings of the present study support the perceptual doping hypothesis by showing a relatively larger gain provided by prior AV speech exposure than by A speech exposure for subsequent improvement in the processing of speech stimuli. In the sentences-in-noise task, however, a procedural learning effect (due to the complexity of speech materials) or a combination of procedural learning and perceptual learning effect (due to the presentation of sentences in background noise) was observed, in addition to the perceptual doping effect. Regarding the clinical relevance of the perceptual doping hypothesis, the authors of the present study suggest that AV speech training could be offered instead of A speech training in the aural rehabilitation of people with hearing loss.

## ACKNOWLEDGMENTS

The authors thank Helena Torlofson, Tomas Bjuvmar, and Wycliffe Yumba, who helped in collecting data; Mathias Hällgren for his technical support; and Olle Eriksson for statistical advice.

## Supplementary Material
