# Supplementary material for: Perceptual Doping: An Audiovisual Facilitation Effect on Auditory Speech Processing, From Phonetic Feature Extraction to Sentence Identification in Noise
Source: Ear Hear. 2019 Feb 27;40(2):312–27. doi: 10.1097/AUD.0000000000000616 (PMC6400397; doi:10.1097/AUD.0000000000000616)
Supplement: Supplementary file 1 [file aud-40-312-s001.docx]

Appendix A: A summary of *t*-test results of multiple comparisons that were used to evaluate the perceptual doping hypothesis by comparing auditory and audiovisual scores between modality orders of presentation of audiovisual first–auditory second (AV1–A2) and auditory first–audiovisual second (A1–AV2)

| Task | Modality order of presentation | Modality  type | *t* test results |
| --- | --- | --- | --- |
| Gated consonants | AV1–A2 vs. A1–AV2 | A  AV | *t*(197) = 6.54, *p* = < .001, *d* = 0.93  *t*(197) = 0.97, *p* = .332 |
| Gated Vowels | AV1–A2 vs. A1–AV2 | A  AV | *t*(197) = 4.62, *p <* .001, *d =* 0*.*66  *t*(197) = 0.13, *p* = .900 |
| Vowel duration discrimination | AV1–A2 vs. A1–AV2 | A  AV | *t*(197) = 4.45, *p* < .001, *d =* 0*.*64  *t*(197) = 1.05, *p* = .295 |
| Sentences in noise | AV1–A2 vs. A1–AV2 | A  AV | *t*(197) = 4.81, *p* < .001, *d =* 0*.*69  *t*(197) = 2.29, *p* = .023, *d* = 0.32 |

Appendix B: A summary of *t*-test results of multiple comparisons that were used to compare audiovisual over auditory presentation on each speech task within each modality order of presentation of audiovisual first–auditory second (AV1–A2) and auditory first–audiovisual second (A1–AV2)

| Task | Modality order of presentation | |
| --- | --- | --- |
|  | AV1–A2 | A1–AV2 |
| Gated consonants | *t*(98) = 5.88, *p* < .001, *d* = 0.59 | *t*(99) = 15.53, *p* < .001, *d* = 1.55 |
| Gated vowels | *t*(98) = 1.68, *p* = .095 | *t*(99) = 5.87, *p* < .001, *d* = 0.59 |
| Vowel duration discrimination | *t*(98) = –3.88, *p* < .001, *d* = 0.39 | *t*(99) = 4.29, *p* < .001, *d* = 0.43 |
| Sentences in noise | *t*(98) = 20.98, *p* < .001, *d* = 2.11 | *t*(99) = 29.62, *p* < .001, *d* = 2.96 |
